# A Biocontrol Strain of *Bacillus subtilis* WXCDD105 Used to Control Tomato *Botrytis cinerea* and *Cladosporium fulvum* Cooke and Promote the Growth of Seedlings

**DOI:** 10.3390/ijms19051371

**Published:** 2018-05-04

**Authors:** Hui Wang, Yuying Shi, Doudou Wang, Zhongtong Yao, Yimei Wang, Jiayin Liu, Shumei Zhang, Aoxue Wang

**Affiliations:** 1 College of Life Sciences, Northeast Agricultural University, 150030 Harbin, China; wh571080005@163.com (H.W.); wangdou123dou@163.com (D.W.); Jessica0504wym@163.com (Y.W.); 2 College of Horticulture and Landscape Architecture, Northeast Agricultural University, 150030 Harbin, China; 1144869388@126.com; 3 Key Laboratory of Biology and Genetic Improvement of Horticultural Crops (Northeast Region), Ministry of Agriculture, Northeast Agricultural University, 150030 Harbin, China; 4 College of Agronomy, Northeast Agricultural University, 150030 Harbin, China; yaozhong123tong@163.com; 5 College of Science, Northeast Agricultural University, 150030 Harbin, China; 13040216@163.com; 6 Institute of Microbiology Heilongjiang Academy of Sciences, 150001 Harbin, China; shumeizhang@yahoo.com

**Keywords:** tomato gray mold, tomato leaf mold, *Bacillus subtilis*, biological control

## Abstract

In this study, a strain named WXCDD105, which has strong antagonistic effects on *Botrytis cinerea* and *Cladosporium fulvum* Cooke, was screened out from the rhizosphere of healthy tomato plants. The tomato plants had inhibition diameter zones of 5.00 mm during the dual culture for four days. Based on the morphological and physiological characteristics, the 16S rDNA sequence, and the *gyrB* gene sequence analysis, the strain WXCDD105 was identified as *Bacillus subtilis* suBap. *subtilis*. The results of the mycelial growth test showed that the sterile filtrate of the strain WXCDD105 could significantly inhibit mycelial growth of *Botrytis cinerea* and *Cladosporium fulvum* Cooke. The inhibition rates were 95.28 and 94.44%, respectively. The potting experiment showed that the strain WXCDD105 made effective the control of tomato gray mold and tomato leaf mold. The control efficiencies were 74.70 and 72.07%. The antagonistic test results showed that the strain WXCDD105 had different degrees of inhibition on 10 kinds of plant pathogenic fungi and the average inhibition rates were more than 80%. We also found that the strain WXCDD105 stimulated both the seed germination and seedling growth of tomatoes. Using the fermentation liquid of WXCDD105 (10^8^ cfu·mL^−1^) to treat the seeds, the germination rate and radicle length were increased. Under the treatment of the fermentation liquid of the strain WXCDD105 (10^6^ cfu·mL^−1^), nearly all physiological indexes of tomato seedlings were significantly higher than that of the control groups. This could not only keep the nutritional quality of tomato fruits but also prevent them from rotting. This study provided us with an excellent strain for biological control of tomato gray mold, tomato leaf mold, and tomato growth promotion. This also laid the technical foundation for its application.

## 1. Introduction

The tomato is native to Peru and Ecuador. It is a solanaceae herbaceous plant that belongs to the solanaceae family. It can be grown for many years under suitable conditions and is one of the most widely grown and highly consumed vegetable crops in the world [1]. Tomato gray mold and leaf mold are the main diseases during tomato cultivation, which is caused by *Botrytis cinerea* and *Cladosporium fulvum* Cooke [2,3]. Gray mold harms tomato leaves, flowers, and fruits on the ground. The flower period and fruit enlargement period suffer the most [4]. The leaf mold disease mainly harms the leaves, but can also infect the stem and fruits. This usually happens in the middle and late stages of production. Because of the lack of resistant germplasm resources and the loss of variety in resistance, the plant has not been screened for ideal tomato gray mold and leaf mold disease resistance resource materials worldwide. The breeding progress of disease resistance is slow. At present, chemical pesticides are still mainly used for preventing and treating tomato gray mold and leaf mold diseases [5,6]. Chemical pesticides have played a huge role in the control of plant diseases, but the disadvantages are also becoming more prominent. Using chemical pesticides for a long time can increase pathogens’ resistance to chemicals. In addition, while it kills germs, it can also threaten beneficial microorganisms, destroy the agricultural ecosystem, and endanger human health. In recent years, biological control has attracted more attention [7]. Biological control can control the disease to the lowest level. Its advantages include low toxicity, low or no residual levels, low pollution, safety, and efficiency. In the background of the sustainable development of agriculture, food safety and environmental protection, the need to research and develop new biological pesticides that will gradually replace chemical pesticides has become an important target for agricultural production. There are many successful cases of the application of antagonistic microorganisms to control tomato gray mold and leaf mold diseases [8,9]. The prophylaxis and treatment of gray mold disease is more than that of leaf mold [10]. According to related reports, the main antagonistic microorganisms of tomato gray mold and leaf mold diseases include bacterial, fungal, yeasts and actinomycetes [11,12]. *Bacillus subtilis* is widely used in biological control as a variety of antibiotics [13]. However, most studies are only screening the microorganism for one of the diseases and the two diseases often occur simultaneously to the actual form of production. The anti-biological inoculant and antimicrobial spectrum of the microorganisms were narrow, which severely restricted its development and application.

Physiological and biochemical characteristics of *Bacillus subtilis*, a kind of gram positive bacterium, have been studied for decades [14,15]. Hundreds of *Bacillus subtilis* have been purified. There are also many kinds of *Bacillus subtilis* that can produce antibiotics. *Bacillus subtilis* can also affect other microbial communities to a certain extent [16]. Therefore it has been increasingly used in the research on biological control.

This study mainly focused on screening new active bacteria and their biocontrol effects. The *Bacillus subtilis* strain WXCDD105 had strong antagonism to the tomato gray mold disease and the leaf mold disease, which were isolated from healthy tomato plants’ rhizosphere soil and identified as *Bacillus subtilis* suBap. *subtilis*. The effects of biological control on other related diseases and its effect on growth was studied. We provided and developed a potential bacterial strain that can prevent tomato gray mold and tomato leaf mold diseases. This also provides a satisfactory technical basis for developing one kind of new biocontrol agent.

## 2. Results

### 2.1. The Separation, Purification, and Screening of Antagonist Strains

**Figure 1:**
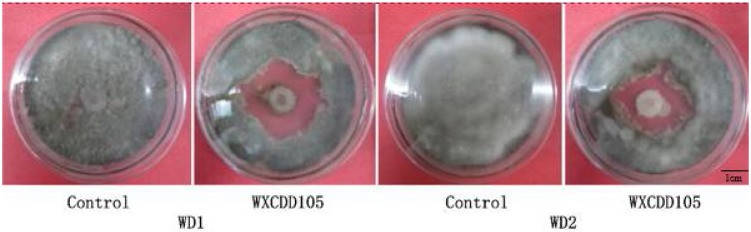
Antagonistic effects of strain WXCDD105 against *Botrytis cinerea* WD1 and *Cladosporium fulvum* Cooke WD2.

A total of 126 strains of bacteria were isolated and purified from rhizosphere soils of different plants. Five antagonistic strains that have different degrees of inhibition effect on *Botrytis cinerea* WD1 and *Myceliophthora* sp. WD2 were obtained by dual-culture screening and were numbered 105, 51, 98, 15, and 109, respectively. The test results of the dual culture are shown in Table 1 below. It can be seen from Table 1 that the strain WXCDD105 isolated from tomato rhizosphere soil has the strongest bacteriostatic activity and the bacteriostatic effects on the strains *Botrytis cinerea* WD1 and *Cladosporium fulvum* Cooke WD2 are, respectively, 71.57 and 71.64%. The antibacterial circle method was used for re-screening and the results are shown in Figure 1. It can be seen from Figure 1 that the strain WXCDD105 has a more stable antibacterial effect on *Botrytis cinerea* WD1 and on *Mycoplasma* WD2. The strain WXCDD105 was, therefore, selected for a follow-up test and it was named WXCDD105.

### 2.2. Identification of Strain WXCDD105

#### 2.2.1. Strain WXCDD105 Morphology Detection

**Figure 2:**
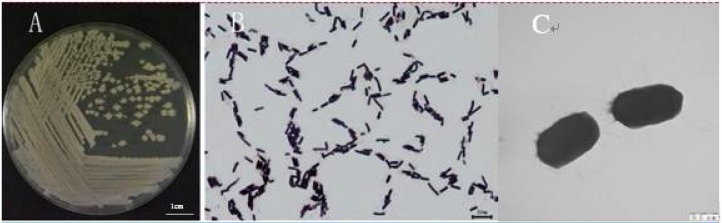
Morphological characteristics of strain WXCDD105 on LB medium plate culture (**A**), and observed under an optical microscopes (**B**) and transmission electron microscopy (**C**).

The colony of strain WXCDD105 was pale yellow, suborbicular, and had a dry surface. It also had a flat, irregular edge, which is shown in Figure 2A after being cultured for 18 h on Luria-Bertani (LB) media at 37 °C. Under optical microscopes, which is shown in Figure 2B, the shape of the bacteria was rod-like and the size of the bacteria was 0.7–0.8 μm × 1.8–3.3 μm. The bacteria were arranged individually or in pairs and appeared as columnar, intermediate, or near-terminal spores whose cyst enlargement was not obvious. They were identified as gram positive. Under a transmission electron microscope, the the bacteria was rod-shaped, round, and had a peri-flagellum, which is shown in Figure 2C.

#### 2.2.2. Molecular Identification of Bacterial Strain WXCDD105

The strain WXCDD105 consists of aerobic bacteria and ferments to produce acid. The nitrate reduction reaction, starch hydrolysis, contact enzyme reaction, gelatin liquefaction, and the citrate utilization test were positive. The compact automatic bacterial identification system of the WXCDD105 strain is shown in Table 2. According to the detection results of Bergey’s Manual of Determinative Bacteriology, the bacterial strain WXCDD105 was identified as *Bacillus subtilis* suBap. *Subtilis*. 

**Figure 3:**
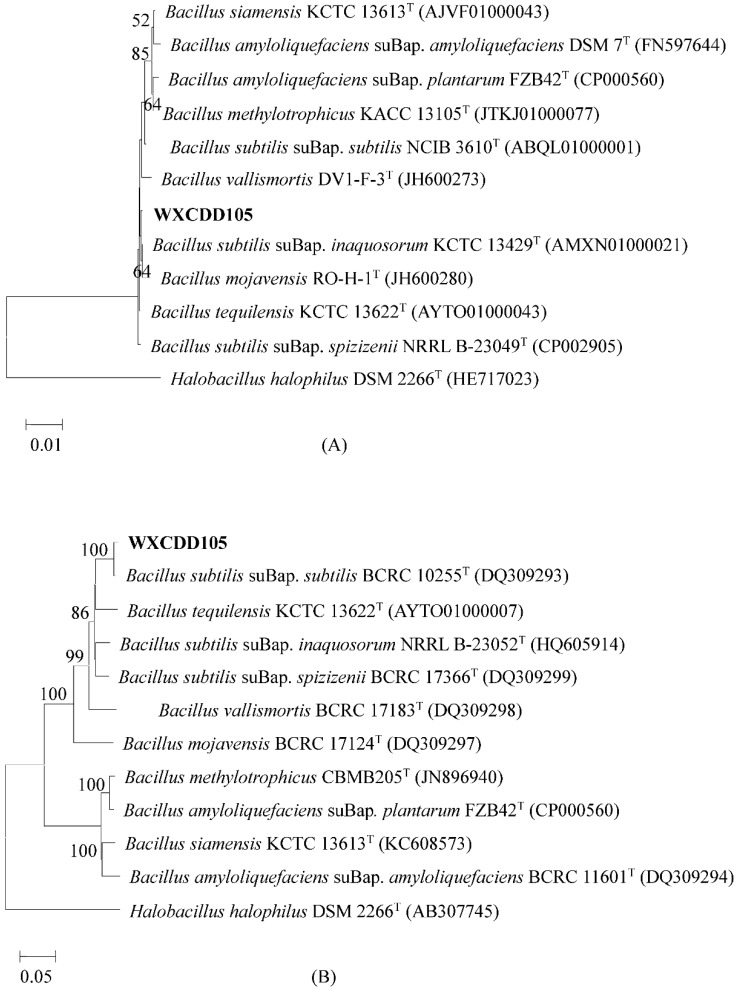
Phylogenetic tree based on 16S rDNA (**A**) and *gyrB* (**B**) sequences of strain WXCDD105. The nodes of the phylogenetic tree where the value of bootstrap is greater than 50%, which will be noted in the graph, and the superscript “T” indicates the model strain.

According to 16S rRNA sequencing results, the most homologous strains of WXCDD105 were *Bacillus* sp., which had the highest similarity with *Bacillus subtilis* suBap. *inaquosorum* (AMXN01000021) and *Bacllus tequilensis* (AYTO01000043). All of these strains were 99.93% similar. The similarity of the *Bacillus amyloedfaciens* suBap. *amylol* (FN597644) in the same branch was 99.34%. In order to improve the species precision, the strain WXCDD105 was analyzed for the *gyrB* gene sequence, the strain WXCDD105 was analyzed for the *gyrB* gene sequence, which is shown in Figure 3.

The *Bacillus subtilis* suBap. *Subtilis* BCRC 10255T (DQ309293) was in the same branch as the strain WXCDD105, which had the highest similarity in 99.1%. The similarity with other model strains was less than 95%. After morphological observation as well as the physiological and biochemical characterization of the 16S rDNA sequence and *gyrB* gene sequence analysis, the strain WXCDD105 was identified as *Bacillus subtilis* suBap. *subtilis*.

### 2.3. Effect of Strain WXCDD105 Aseptic Filtrate on the Growth of Mycelia of Botrytis cinerea and Fuliva fulva

**Figure 4:**
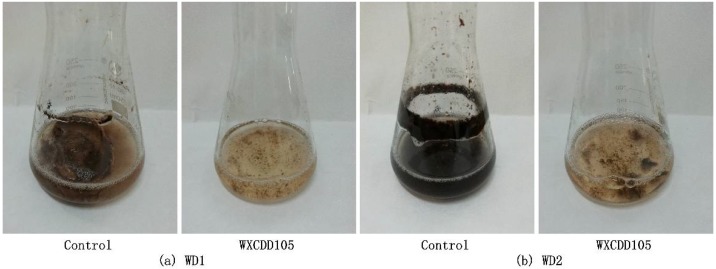
Inhibitory effects of sterile filtrate of strain WXCDD105 on mycelial growth of *Botrytis cinerea* WD1 (**a**) and *Cladosporium fulvum* Cooke WD2 (**b**).

The bacterial strain WXCDD105 aseptic filtrate showed a significant inhibitory effect on the growth of the pathogens of tomato gray mold disease and tomato leaf mold disease. The hypha of *Botrytis cinerea* WD1 and the *Cladosporium fulvum* Cooke WD2 grow rarely in the strain WXCDD105 solution in contrast to the ddH_2_O solution as a control, which is shown in Figure 4. The inhibitory rate of strain WXCDD105 on the growth of *Botrytis cinerea* WD1 mycelia and the growth of *Cladosporium fulvum* Cooke WD2 mycelia was 95.28 and 94.44%, respectively. Additionally, there was no significant difference between them, which is shown in Table 3 below. The results indicated that the strain WXCDD105 can produce certain bacteriostatic substances and inhibit the growth of the tomato gray mold and leaf mold pathogens.

### 2.4. The Biocontrol Effect of Strain WXCDD105 on Tomato Gray Mold and Tomato Leaf Mold

**Figure 5:**
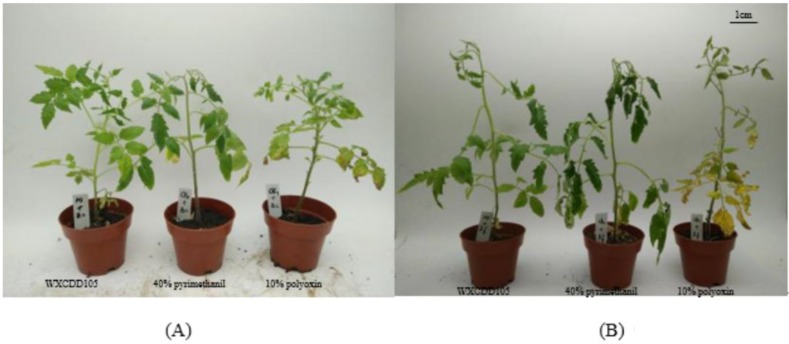
The biocontrol effects of tomato gray mold disease (**A**) and tomato leaf mold disease (**B**) infected tomato leaves.

A healthy tomato seedling grown to five real leaves was tested for efficacy. It was set for three repetitions with 10 seedlings repeating in temperature 25 °C and humidity 90% condition culture. After seven days of inoculation, the disease was observed day by day and the disease index and control effect were calculated. After seven days of inoculation of mildew and leaf mold, the incidence of individual leaves was increased and the number of leaves was gradually increased after 10 days. The symptoms on day 15 were obvious. As shown in Figure 5A,B. At this time, the disease index of tomato gray mold and leaf mold diseases were 74.70 and 72.07%, respectively. In addition, compared to the control of ddH_2_O, the disease index by chemical pesticides and the strain WXCDD105 treatment was significantly lower. The protective effect of 40% pyrimethanil (chemical) on the tomato gray mold disease was 67.77% and the protective effect of 10% polyoxin (chemical) on the tomato leaf mold disease was 70.09%. WXCDD105 has a good biocontrol effect on *Botrytis cinerea* and *Cladosporium fulvum* Cooke simultaneously, and the control effects were respectively 74.70% and 72.07%, which are better than chemical pesticides of 40% ethyl. Pyrimethanil (preventing gray mold) and 10% polyoxin (preventing leaf mold), as shown in Table 4.

### 2.5. Determination of Bacteriostasis Spectra of Strain WXCDD105

The results of the antibacterial spectrum determination are shown in Table 5. It can be observed from this table that the bacteriostasis spectrum of strain WXCDD105 is wide. The strain WXCDD105 has an inhibitory effect on 10 plant pathogenic fungi, which cause root and leaf diseases of crops. Among them, the inhibition rate of *Botrytis cinerea* was the highest at 97.37%. The inhibition rate of the *Fusarium oxysporum* f. sp. *niveum* disease was the lowest at 77.45%. The inhibition rate of other diseases was between 77 and 95%.

### 2.6. Growth-Promoting Effects of Strain WXCDD105

**Figure 6:**
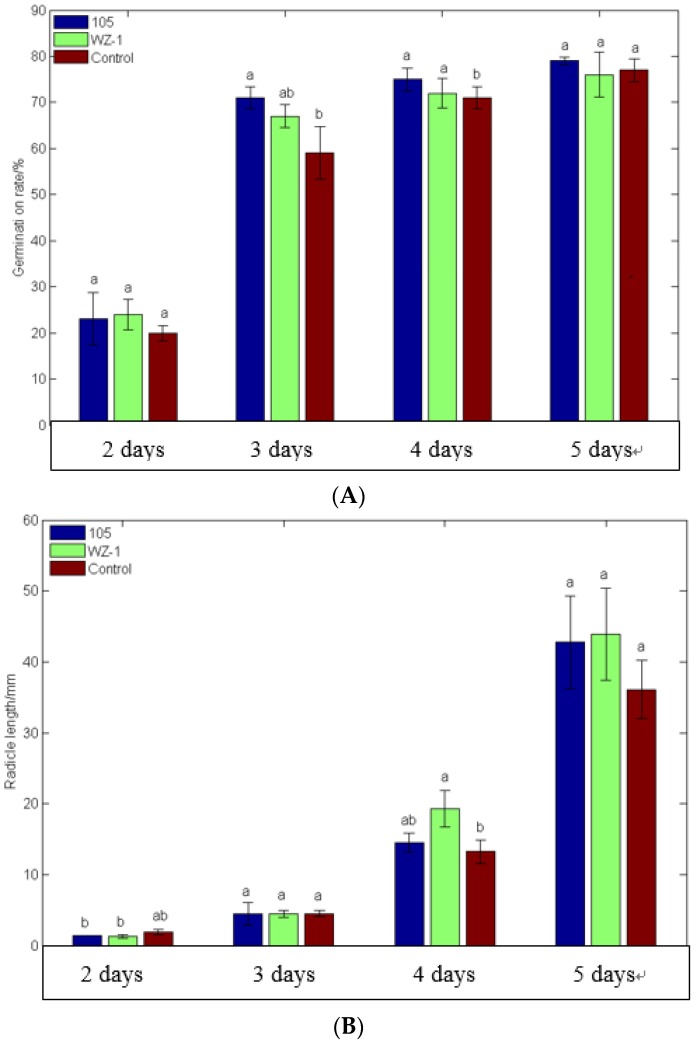
Effects of the strain WXCDD105, the strain WZ-1 and the water control on seed germination (**A**) and radicle growth (**B**) of tomato. The different normal letters in the same point indicate significant difference among treatments at 0.05 level (*n* = 3).

Tomato seeds were soaked in the strain WXCDD105 with a concentration of 10^8^ cfu·mL^−1^ for 3 h. The germination rate and the root growth of the radicle were measured and the result is shown in Figure 6. The strain WXCDD105 can promote the growth of tomato seedlings to a certain extent. The germination rate of tomato seedlings which were treated with the strain WXCDD105 was 79.00% at the fourth day after treatment, while the germination rates of tomato seedlings treated with the *Bacillus subtilis* WZ-1 and clear water were 72.00 and 71.00%, respectively, during the fourth day after treatment. The strain WZ-1 was isolated in our lab. The effects of three treatments of the radicle length of the sprouting tomato had no significant difference in the first three days. But during the fourth day, the radicle lengths of the tomato seed soaked with strain WXCDD10, WZ-1, and water were 14.52, 19.34, and 13.26 mm, respectively. During the fifth day, the radicle lengths of the tomato seed soaked with the strain WXCDD10, WZ-1, and water were 42.78, 43.94, and 36.08 mm, respectively. The radicle length of tomato seeds soaked with the strain WXCDD10 increased 18.57% over the control group soaked with water and was not significantly different to the control group soaked with strain WZ-1.

Table 6 below shows the effect of the strain WXCDD105 on the growth promotion of tomato seedlings. It can be seen from the table that different concentrations of strain WXCDD105 have certain effects on the growth of tomato seedlings at the 4–5 leaf stage. In addition, the bacterial liquid with a concentration of 10^6^ cfu·mL^−1^ had the best promoting effect on tomato seedlings. The plant height, stem diameter, main root length, and whole plant fresh weight of tomato seedlings treated with bacteria liquid with a concentration of 10^7^ cfu·mL^−1^, 10^6^ cfu·mL^−1^ were significantly higher than the control. Compared with the water control group, the plant height, stem diameter, main root length, and whole plant fresh weight of tomato seedlings treated by bacterial liquid with a concentration of 10^6^ cfu·mL^−1^ increased by 66.34, 41.82, 112.33, and 145.22%, respectively. Additionally, there were differences between different concentrations of bacterial liquid.

### 2.7. The Effect of Strain WXCDD105 on the Physiological Quality of Tomato Fruit

**Figure 7:**
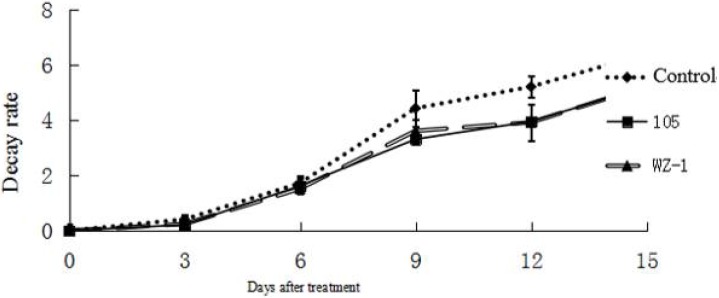
Effects of different treatments on weight-loss rate of tomato.

With the extension of storage time, there was not much difference between the changes in storage from three to nine days. The mass loss rate increased sharply and the control increased significantly. However, the quality loss of fruit treated by strain WXCDD105 was lower than the control (Figure 7). After 15 days of storage, the rotten rate of processing the tomato fruit treated by strain WXCDD105 bacterial suspension was significantly lower than the control group. The strain WXCDD105-treated decay rate was 29.45% and the biocontrol bacteria strain WZ-1 decay rate was 38.14%, while the control group had a decay rate of 57.07% (Table 7). The strain WXCDD105 has a superior antiseptic effect and strong effects on storage of the biocontrol bacterium WZ-1. After 15 days of storage, the control fruit became soft compared with the biocontrol bacteria strain WXCDD105, and WZ-1-treatment fruit firmness was 0.68, 0.70, and 0.56 kg·cm^−2^, respectively. The biocontrol agent strain WXCDD105 can delay fruit softening, but has no significant difference with the biocontrol agent strain WZ-1. At the end of storage for different groups of soluble solids, the titratable acid content was determined. The results showed that there was no significant difference in the nutrient content between the treatments (see Table 7).

## 3. Discussion

In recent years, the use of *Bacillus subtilis* and biological control of the plant have been carried out extensively [17]. In these studies, some strains can produce Idole acetic acid (IAA) and some strains can produce iron cells [18,19]. The *Bacillus* strains can significantly inhibit the growth of pathogenic bacteria and pathogenic bacteria biofilm synthesis of the cell wall that will cause severe damage to plants and lead to cell lysis of pathogens. Additionally, the interaction of rhizosphere bacteria and plants had the effect of promoting plant growth [20]. Previous research showed that *Bacillus subtilis* during the process of phosphorylation has an ability to dissolve inorganic phosphate and can increase the utilization rate of phosphorus in the soil. *Bacillus* resistance can lead to the secretion of a spectrum of the compounds or the induced resistance of plants to increase system capacity [21]. *Bacillus* produces siderophores at the same time in order to adhere to the surface of non-biological biofilm formation, which makes the *Bacillus* secrete broad-spectrum resistance compounds to a certain extent and increase the spectrum resistance of plants [22]. The tomato plant has attracted increasing attention because of its special flavor, rich vitamins, and its excellent antioxidation effects. Tomato plants are planted in many areas in China but are vulnerable to many diseases such as fungi, bacteria, and nematodes both in the greenhouse and field [23,24]. Tomato gray mold and tomato leaf mold diseases are the main causes of large area reductions in tomato cultivation. These two diseases have mainly occurred in the aboveground parts of plants, which seriously threatens plant growth and development [25]. Boukaewet reported that *Streptomyces* is a genus known for its ability to protect plants against many pathogens and various strains of this bacteria have been used as biological control agents [26]. At present, the prevention and treatment of these two diseases are mainly based on chemical control, but the long-term use of chemical fungicides will lead to food safety, environmental pollution, and pathogenic bacterial resistance. A series of problems such as a single control effect has serious harm. A growing number of researchers are paying attention to these problems. More people began to use biological control methods in the control of disease and made great progress. Biocontrol agents mainly *Bacillus*, *Trichoderma*, *Pseudomonas*, nonpathogenic *Fusarium*, and *Penicillium* strains were evaluated to control *Fusarium* wilt, but still this lethal disease could not be controlled completely [23]. Biological or biological metabolites provide effective protection for animal and plant control. The biggest feature of this form of protection is not the fact that it will not destroy the natural environment and will not hurt people, animals, and plants, but also that it will not pollute or destroy the normal natural environment and has the advantages of controllability and stability. In recent years, the application of *Bacillus* spore to control plant diseases has been reported worldwide. The application includes the effect of *Bacillus subtilis* on the growth immune function and intestinal morphology in weaned piglets. The present study compared the effects of soybean meal fermented by three different probiotics organisms with a non-fermented soybean meal on the growth performance, serum parameters, immune chemistry, and intestinal morphology of weaned piglets [23]. The *Bacillus subtilis* strain WXCDD105 obtained in this study can prevent *Corynesporacassiicola*, *Botrytiscinerea*, *Sclerotiniasclerotiorum*, *Setosphaeria turcica*, among others. The control effect is more than 80%. In particular, the effect of the prevention of gray mold was 10.26% higher than that of 40% Pyrimethanil. Additionally, it was higher than for the control of tomato leaf mold by 10% Polyoxin. In this study, 126 strains of bacteria were isolated from soil. Five of them were 105, 51, 98, 15, and 109, which had some control effect of *Botrytis cinerea* and *Cladosporium fulvum* Cooke, while 105 had the best control effect against both diseases. The mechanism of *Bacillus subtilis* disease prevention has been studied. Asaka reported that *Bacillus subtilis* secreted antibiotics iturin A and surfactin for controlling plant diseases [27]. *Bacillus subtilis* enhanced the expression of the chitinase promoter [28]. Such bacteria indicated expression of both chiS and chiL chitinases under the control of a strong promoter. The activity of this promoter was evaluated in *B. subtilis* as a host organism. Our study found that 105 of the aseptic filtrate had a significant control effect on tomato gray mold and tomato leaf mold mycelia growth and all of them reached over 90%. This also indicates that these 105 can produce some antibacterial substances during the growth process, which stops plant diseases. Al-Masri studied this in only 73% of cases [29].

On the general antibacterial range, especially in terms of the *Botrytis cinerea* and tomato *Cladosporium fulvum* Cooke, the significant control effect not only showed that strain WXCDD105 has a certain promoting effect on tomato seeds and seedlings, but also the characteristics necessary to maintain the quality of the tomato fruit. The firmness compared to the control group increased 21.42% and greatly reduced the decay rate of the tomato. However, tomato firmness, soluble solids content, and titratable acid content had no significant difference when compared to those with water in the control group. Fresh weight may be the cause of the seedling test, where there is no big difference; but as the plant grows, there may be large differences. We also found that the biocontrol strain WZ-1-treated tomato fruit reached the approximate treatment effect. Whether the biocontrol affected the tomato fruit and produced a similar biocontrol substance, and whether this is the source of plant-induced disease resistance, this is how the role of the antimicrobial substance in the tomato induced the overexpression of the tomato gene and its mechanism of action. This mechanism needs to be studied further.

## 4. Materials and Methods

### 4.1. Materials

Pathogens *Botrytis cinerea* WD1, *Cladosporium fulvum* Cooke WD2, *Corynespora cassiicola* HH, *Botrytis cinerea* WS1, *Sclerotinia sclerotiorum* WS2, *Setosphaeria turcica* YD, *Fusarium oxysporum* f. sp. *cucumerinum* WS3, *Fusarium oxysporum* f. sp. *niveum* XK, *Septoria lycopersici* Speg. FB, *Fusarium oxysporium* Schelcht SL, *Fusarium oxysporum* f. sp. *lycopersici* FK, and *Fusarium oxysporum* f. sp. *melonis* TK were kept and propagated at 25 °C on potato dextrose agar (PDA) plates in our lab.

Additionally, 126 strains of bacteria were isolated from the rhizosphere soil of a carrot, *Portulaca*, *Solanum nigrum*, a radish, wheat, soybean, Chinese cabbage, and a tomato. The biocontrol bacteria strain WXCDD105 was screened for 126 strains and originated from tomato rhizosphere soil. *Bacillus* sp. X was maintained in our lab. Previous studies have shown that it provided effective control of tomato gray mold, promoted tomato plant growth, and had a preservation effect. All bacteria were cultured at 37 °C on LB medium.

The tomato cv. Dongnong 713 was provided by the Tomato Research Institute of Northeast Agricultural University in China. It has no resistance to *Botrytis cinerea* and *Cladosporium fulvum* Cooke. The plants were grown from seeds in growth chambers at 25/22 (day/night), 75% relative humidity, and a 16/8 h (day/night) photoperiod. Healthy tomato fruit and leaves with similar sizes and colors were used in this study. The fruit and leaves were free from visible wounds and diseases. Before each treatment, the fruit was washed with water and then in 75% ethanol for 30 s, rinsed in sterile water, and air dried.

### 4.2. Isolation and Screening of Antagonistic Bacteria

Soil samples were collected from different fields. The sterile water was mixed with one gram of soil samples with 9 mL of mixing and dilution (10^−1^–10^−6^). 100 µL of sample from 10^−6^ dilution were plated and spread separately onto LB medium and incubated at 37 °C for 24 h. The same morphology, color, and size of colonies was picked up from the LB medium.

The pathogenic fungi (*Botrytis cinerea* WD1 and *Cladosporium fulvum* Cooke WD2) were inoculated on potato dextrose agar (PDA) medium for 1 week. A mycelia disk (7 mm diameter) of each pathogen was transferred to the center of the freshly prepared PDA medium on different plates and the bacteria were inoculated onto the areas 20 mm from the central pathogen colony, which was followed by incubation at 28 °C for 7 days. Each experiment was replicated three times and the average zone of inhibition was calculated.

Antagonistic strains were screened repeatedly by using the inhibition zone method. 50 µL of the spore suspension of *Botrytis cinerea* WD1 and *Cladosporium fulvum* Cooke WD2 (10^5^ cfu·mL^−1^) were plated and spread separately onto PDA medium and incubated at 25 °C for 14 days. The Oxford cup was transferred to the center and the antagonistic bacteria fermentation broth was added to the Oxford cup. Each experiment was replicated three times and the average zone of effects was measured.

### 4.3. Morphological and Biochemical Characteristics of Strain WXCDD105

According to Bergey’s Manual of Determinative Bacteriology [12], the morphological characteristics and physiological and biochemical characteristics of the strain WXCDD105 were identified by conventional methods.

Genomic DNA extraction of strain WXCDD105 was performed as described by the CTAB method and PCR amplification of the 16S rRNA gene was carried out with universal primers (8F: 5′-AGAGTTTGATCMTGGCTCAG-3′, 1492R: 5′-ACGGATACCTTGTTACGACTT-3′). The conditions used for the thermal cycler (TaKaRa, Dalian, China) were measured according to the following amplification profile: 94 °C for 3 min followed by 30 cycles consisting of denaturation at 94 °C for 30 s, primer annealing at 55 °C for 30 s and primer extension at 72 °C for 1 min. At the end of the cycles, the reaction mixture was kept at 72 °C for 10 min and then cooled to 4 °C. PCR amplification of the *gayB* gene was carried out with universal primers (UP-1: 5′-GAAGTCATCATGACCGTTCTGCAYGCNGGNGGNAARTTYGA-3′, UP-2r: 5′-AGCAGGGTACGGATGTGCGAGCCRTCNACRTCNGCRTCNGCRTCNGTCAT). The conditions used for the thermal cycler (TaKaRa) were based on the following amplification profile: 96 °C for 5 min followed by 30 cycles consisting of denaturation at 94 °C for 30 s, primer annealing at 63 °C for 90 s, and primer extension at 73 °C for 90 s. At the end of the cycles, the reaction mixture was kept at 72 °C for 10 min and then cooled to 4 °C. The extracted product was recovered and purified by a gel recovery kit and was sequenced by the Beijing Genomics Institute (BGI) (Beijing, China). The 16S rRNA and *gayB* gene sequences determined were compared with the GenBank database by using the BLAST search program. The phylogenetic and molecular evolutionary analyses were conducted using the software included in the MEGA 6.06 package. The 16S rRNA and *gyrB* gene sequences of the strain WXCDD105 and 11 types of model strains were aligned using the ClustalX 1.83 program [30]. The evolutionary tree was inferred by using the neighbor-joining method [31] from the evolutionary distance data corrected by Kimura’s two-parameter model [32], and the topology of the phylogenetic tree was evaluated by the bootstrap resampling method of 1000 replicates [33].

### 4.4. The Inhibiting Effect of the Biocontrol Bacterium WXCDD105 Fermentation Filtrate on the Growth of Tomato-Grown Mold and Leaf Mold

We transferred the bacterial strain WXCDD105 to LB broth, 30 °C, 200 rpm shake culture for 72 h, the 10,000 rpm centrifuge for 10 min, and took on a clear liquid with 0.22 μm aseptic filtrate microporous membrane filter. The strain WXCDD105 aseptic filtrate was added to the same amount of aseptic 2-fold concentrated PDA medium to produce the medium. Afterward, 50 mL containing medium was added to 250 mL triangular bottles and inoculated with two strains of *Botrytis cinerea* WD1 and *Cladosporium fulvum* Cooke WD2 (7 mm in diameter). This was maintained in sterile water for contrast, trained for 5 days in the condition of 28 °C, 120 rpm, filtered by gauze, and then the hyphae were collected, weighed, and the inhibition rate calculated. Each process was repeated three times.

### 4.5. Antifungal Activity Assay Using Strain WXCDD105 in the Greenhouse

Tomato seeds were surface-sterilized for 15 min in 2% sodium hypochlorite followed by a brief rinse with 75% ethanol. Seeds were sown on a seedling tray filled with commercially available horticulture soil. The soil used in the experiment was steam-sterilized at 121 °C for 30 min three times. The seedlings were grown in the greenhouse. After four weeks, individual seedlings were transplanted into plastic pots (10 cm in diameter) and filled with the same sterile soil. Plants were watered twice a day. Plants were maintained at 20–30 °C in the greenhouse for two months. *Botrytis cinerea* WD1, *Cladosporium fulvum* Cooke WD2 were cultured on a PDA plate at 20 °C for two weeks. A conidial suspension was diluted in potato dextrose broth after incubation to reach the final concentration of 10^5^ cfu·mL^−1^, which was determined with a hemocytometer. Tomato seedlings were treated with the fermentation liquid of strain WXCDD105 (10^8^·cfu·mL^−1^) for three consecutive weeks (once a week) by spray inoculation. Then *Botrytis cinerea* WD1 and *Cladosporium fulvum* Cooke WD2 were inoculated. Pyrimethanil (40% wettable powder) and polyoxin (10% wettable powder) were used as positive controls for biocontrol and fungicide effects, respectively, and water was used as the negative control and applied at the same time as the antagonists. The inoculated plants were kept in a moist chamber and maintained at approximately 90% humidity at 25 °C. Disease severity was evaluated at 14 days after inoculation on a scale of 0 to 4. Level 0 stands for no disease, level 1 represents morbidity ≤ 25%, level 2 represents morbidity 25.1–50%, level 3 represents morbidity 50.1–75%, level 4 represents morbidity ≥75.1%. There were three replications for each treatment. All 30 plants per treatment were used for disease symptom investigation. The plant disease index (DI), which would represent both disease incidence and symptom severity, can be calculated as: DI = (ΣDi × Dd)/(Mi × Md) × 100 where i means a 0–4 disease level and Mi means plant number of reaction i, Dd means total number of leaves investigated.

### 4.6. Determination of Biocontrol Bacterial Antibacterial Spectrum

The antimicrobial activity of the bacterial strain WXCDD105 on 10 plant pathogenic fungi was determined by using the mycelial growth rate method. We took 100 μm bioantibacterial fluid (10^8^ cfu·mL^−1^) on the PDA plate and inoculated the pathogenic bacteria cake (7 mm in diameter) at the center of the tablet with the sterile LB medium as the control at 28 °C. When the pathogens in the control group were covered the whole petri dish, we measured the colony diameter and calculated the inhibition rate. Each experiment was repeated three times. Bacteriostatic rate (%) = [(control group pathogenic bacteria diameter − experimental group pathogenic bacteria diameter)/(control group pathogenic bacteria diameter − 7)] × 100.

### 4.7. Effects of Biocontrol Bacteria on the Growth of Tomato Seeds and Seedlings

Same-size grain tomato seeds were selected and added to 2% sodium hypochlorite (*v*/*v*) for 15 min, 75% alcohol, 30 s treatment, then washed in a strain WXCDD105 bacterial liquid of 10 mL after washing 3 times in sterile water (concentration of 10^8^ cfu·mL^−1^). After soaking for 3 h, they were dried and placed in a sterile Petri dish. The filter paper held 50 grains per dish and included 5 dishes. The cultivation of the moisturizing box was maintained at a temperature of 25 °C and water treatment, and the strain WZ-1 was used as the control. Records of the tomato seed germination rate and radicle length were reported at 2–5 days. Each treatment was repeated 3 times.

Tomato seeds were seeded in plantlets containing sterilized soil and managed in a greenhouse after disinfection. Tomato seedlings with the same growth were selected after 10 days of seeding and were treated with strain WXCDD105 liquid, respectively. 10^6^ cfu·mL^−1^ mixed soil was treated with clear water as the control. The optimum concentration was screened and seed soaking was used. After soaking tomato seeds with the best dilution ratio of bacteria for 3 h, the seeds were seeded on a seedling tray until the seedlings grew to 2 leaves and 1 heart. This was transplanted into a nutritious bowl. Soaking seeds in sterile water was used as a control. Seeds were disinfected and seeded in a seeding tray. Uniformly grown seedlings were positioned in the pot with the best dilution broth with root treatment. Each bowl held 20 mL and sterile water irrigation treatment was used as a control. Seed disinfection after sowing to seedling trays was used to grow to 2 leaves and 1 heart. Uniform grown seedlings were transplanted into pots with each bowl containing an equal amount of nutrient soil mixed with 20 mL optimum dilution of bacteria liquid with sterile water and soil treatment used as a control. After the completion of each treatment in the greenhouse under routine management, when the plants reached the 4–5 leaf stage they underwent a determination of the plant height, stem diameter and root length.

### 4.8. The Effects of Bacteria Strain WXCDD105 on the Physiological Quality of Tomato Fruits

The fruits of the tomato that had no damage and disease with the same size maturity were graded, weighed and washed with sterile water. They were soaked for 4 min in strain WXCDD105 bacterial suspension liquid (10^7^ cfu·mL^−1^). In addition, they were dredged to dry and store in a fresh bag at room temperature. The condition of being putrefied was observed and weighed every 3 days and the weight loss rate was calculated. After the storage period, some indexes were tested including firmness, soluble solid, and titratable acid content. The test was treated and contrasted with aseptic water and the antibacterial X was treated as a positive control. In total, 30 fruits were processed in each group and each treatment was repeated 3 times.

Fruit rot rate: fruit rot rate of tomato (%) = rotten number of fruits/total number of fruits × 100, Weight loss rate: fruit weightlessness (%) = (initial fruit weight − survey fruit weight)/initial fruit weight × 100. Determination of the firmness of the fruit: using the GY-4 fruit firmness tester to record readings (Zhejiang Top Instrument Co., LTD., Hangzhou, China). Determination of soluble solids: using a hand-held refractometer to record readings. Titration acid was determined by acid base titration.

Excel 2013 (Microsoft) software was used to carry out the statistical analysis, DPS (Data processing system) V15.10 (Hangzhou Ruifeng Information Technology Co. LTD., Hangzhou, China) to carry out data analysis, and Duncan’s test was performed. 

## 5. Conclusions

We isolated a bacterium that had a good control effect on *Botrytis cinerea* and *Cladosporium fulvum* Cooke from the rhizosphere soil of a healthy plant and identified it as *Bacillus subtilis*. The strain not only showed significant potential for biological control but also promoted the growth of tomato seedlings.

## Figures and Tables

**Table 1:** Screening of biocontrol bacteria against tomato gray mold disease and tomato leaf mold disease.

Strain Number	*Botrytis cinereal* WD1		*Cladosporium fulvum* Cooke WD2	
	Inhibition Zone/mm	Inhibition Rate/%	Inhibition Zone/mm	Inhibition Rate/%
105	4.99 ± 0.56 a	71.57 a	5.01 ± 0.75 a	71.64 a
51	4.00 ± 0.63 a	69.13 a	4.00 ± 0.51 a	69.13 a
98	0.93 ± 0.12 c	61.56 c	0.92 ± 0.14 c	61.53 c
15	1.61 ± 0.41 b	63.23 b	1.62 ± 0.32 b	63.26 b
109	1.00 ± 0.11 a	61.74 a	1.00 ± 0.11 a	61.74 a

Notes: the different normal letters in the same column indicate significant difference among treatments at 0.05 level (*n* = 3).

**Table 2:** The results of the bacterial automatic identification system of the strain WXCDD105.

The Bacterial Strain WXCDD105							
Identification Index	Result	Identification Index	Result	Identification Index	Result	Identification Index	Result
BXYL	+	LysA	−	AspA	−	LeuA	+
PheA	−	ProA	−	BGAL	+	PyrA	+
AGAL	+	AlaA	−	TyrA	−	BNAG	−
APPA	+	CDEX	−	dGAL	−	GLYG	−
INO	+	MdG	+	ELLM	−	MdX	−
AMAN	−	MTE	+	GlyA	+	dMAN	+
dMNE	+	dMLZ	−	NAG	−	PLE	+
lRHA	−	BGLU	+	BMAN	−	PHC	−
PVATE	+	AGLU	−	dTAG	−	dTRE	+
INU	+	dGLU	+	dRIB	+	PSCNa	−
NaCl 6.5%	+	KAN	−	OLD	−	ESC	+
TTZ	+	POLYB_R	−				

Notes: “+” means masculine, “−” means feminine.

**Table 3:** Inhibitory effects of sterile filtrate of strain WXCDD105 on mycelial growth of pathogenic fungi.

Treatment	*Botrytis cinerea* WD1		*Cladosporium fulvum* Cooke WD2	
	Mycelium Dry Weight/g	Inhibition Rate/%	Mycelium Dry Weight/g	Inhibition Rate/%
WXCDD105	0.024 ± 0.009	95.28	0.029 ± 0.006	94.44
Control	0.508 ± 0.014	-	0.522 ± 0.025	-

**Table 4:** The control effects of strain WXCDD105 against tomato gray mold disease and tomato leaf mold disease on seedlings in the pot experiment.

Treatment	Tomato Gray Mold Disease		Tomato Leaf Mold Disease	
	Disease Index	Control Efficiency/%	Disease Index	Control Efficiency/%
WXCDD105	10.73 ± 2. 41 b	74.70	12.09 ± 2.89 b	72.07
40% Pyrimethanil	13.67 ± 2.63 b	67.77	-	-
10% Polyoxin	-	-	12.95 ± 2.74 b	70.09
Control	42.41 ± 1.58 a	-	43.29 ± 1.53 a	-

Notes: the different normal letters in the same column indicate significant difference among treatments at 0.05 level (*n* = 3).

**Table 5:** Inhibitory effect of strain WXCDD105 on plant pathogenic fungi.

Pathogenic Fungi	Colony Diameter (mm)		Inhibition Rate/%
	Control	Treatment	
*Corynespora cassiicola*	88.13 ± 0.78	11.49 ± 0.25	94.47 ab
*Botrytis cinerea*	88.56 ± 0.99	9.13 ± 0.42	97.37 a
*Sclerotiniasclerotiorum*	88.51 ± 0.55	16.79 ± 0.25	87.99 c
*Setosphaeria turcica*	88.36 ± 2.70	13.24 ± 1.00	92.33 bc
*Fusarium oxysporum* f. sp. *cucumerinum*	88.83 ± 0.38	16.17 ± 1.72	88.79 c
*Fusarium oxysporum* f. sp. *niveum*	88.33 ± 3.85	25.34 ± 5.68	77.45 d
*Septoria lycopersici Speg.*	88.15 ± 0.45	14.86 ± 0.49	90.31 bc
*Fusarium oxysporium Schelcht*	88.11 ± 1.57	23.98 ± 3.18	79.07 d
*Fusarium oxysporum* f. sp. *lycopersici*	88.89 ± 2.15	16.04 ± 0.02	88.96 c
*Fusarium oxysporum* f. sp. *melonis*	88.50 ± 1.57	22.41 ± 0.83	81.08 d

Notes: the different normal letters in the same column indicate significant difference among treatments at 0.05 level (*n* = 3).

**Table 6:** The growth-promoting effect of single strain WXCDD105 on tomato seedlings.

Processing Method	Plant Height/cm	Stem Diameter/mm	Main Root Length/cm	Whole Plant Fresh Weight/g
10 times dilution	15.30 ± 0.72 b	3.21 ± 0.04 b	13.13 ± 1.78 b	3.52 ± 0.91 b
100 times dilution	17.30 ± 1.31 a	3.90 ± 0.14 a	17.90 ± 1.84 a	5.64 ± 0.14 a
Control	10.40 ± 0.85 c	2.75 ± 0.09 c	8.43 ± 1.81 c	2.30 ± 0.84 b

Notes: the different normal letters in the same column indicate significant difference among treatments at 0.05 level (*n* = 3).

**Table 7:** Effects of different treatments on fruit quality of tomato.

Treatment	Rotting Rate/%	Firmness/kg·cm^−2^	Soluble Solids Content/%	Titratable Acid Content/%
Control	56.67 ± 1.21 a	0.57 ± 0.06 b	6.24 ± 0.21 a	0.32 ± 0.13 a
WZ-1	38.34 ± 1.13 b	0.71 ± 0.02 a	6.25 ± 0.13 a	0.33 ± 0.04 a
WXCDD105	28.15 ± 1.02 c	0.71 ± 0.03 a	6.27 ± 0.22 a	0.35 ± 0.06 a

Notes: the different normal letters in the same column indicate significant difference among treatments at 0.05 level (*n* = 3).

## Abbreviations

WD1	*Botrytis cinerea*
WD2	*Cladosporium fulvum* Cooke
LB	Luria–Bertani
PDA	Potato dextrose agar
PDB	Potato dextrose broth
DI	Disease index
BXYL	Beta-XYLOSIDASE
LysA	L-Lysine-ARYLAMIDASE
AspA	Aspartic-ARYLAMIDASE
LeuA	Leucine-ARYLAMIDASE
PheA	Phenylalanine ARYLAMIDASE
ProA	Proline ARYLAMIDASE
BGAL	BETA-GALACTOSIDASE
PyrA	Pyrrolydonyl ARYLAMIDASE
AGAL	ALPHA-GALACTOSIDASE
AlaA	Alanine ARYLAMIDASE
TyrA	Tyrosine ARYLAMIDASE
BNAG	BETA-N-ACETYL-GLUCOSAMINIDASE
APPA	Ala-Phe-Pro-ARYLAMIDASE
CDEX	Alanine-phenylalanine-proline ARYLAMIDASE
dGAL	D-GALACTOSE
GLYG	Glycogen
INO	Inositol
MdG	Methyl glucoside acidification
ELLM	ELLMAN
MdX	Methyl-d-xyloside
AMAN	Alpha mannosidase
MTE	Germination three sugar
GlyA	Glycine ARYLAMIDASE
dMAN	D-MANNITOL
dMNE	D-MANNOSE
dMLZ	D-MELEZITOSE
NAG	N-ACETYL-GLUCOSAMINE
PLE	PALATINOSE
IRHA	L-rhamnose monohydrate
BGLU	BETA-GLUCOSIDASE
INU	Inulin
dGLU	D-GLUCOSE
dRIB	Ribose
PSCNa	Decomposing ammonia assimilation
OLD	Old sugar
ESC	ESCULIN hydrolyse
TTZ	Polymyxin B resistance
POLYB_R	Polymyxin B tolerance
